# Efficacy of weekly docetaxel in locally advanced cardiac angiosarcoma

**DOI:** 10.1186/s13104-015-1296-4

**Published:** 2015-07-30

**Authors:** Santino Minichillo, Maria Abbondanza Pantaleo, Margherita Nannini, Fabio Coccolo, Lidia Gatto, Guido Biasco, Giovanni Brandi

**Affiliations:** Department of Specialized, Experimental and Diagnostic Medicine, Sant’Orsola-Malpighi Hospital, University of Bologna, Via Massarenti, 9, 40138 Bologna, Italy; “Giorgio Prodi” Cancer Research Center, University of Bologna, Via Massarenti, 9, 40138 Bologna, Italy; Cardiovascular Department of the University of Bologna, Via Massarenti, 9, 40138 Bologna, Italy

**Keywords:** Angiosarcoma, Soft tissue sarcoma, Taxanes, Docetaxel, Cardiac tumors

## Abstract

**Background:**

Primary cardiac angiosarcoma is extremely aggressive; however, it is often misdiagnosed because of its rarity. For locally advanced tumors, doxorubicin-based chemotherapy regimens are the standard of treatment, even if the gain in term of progression-free survival is limited and is no longer than 5 months.

**Case presentation:**

We report the case of a Caucasian 23-year-old man with locally advanced cardiac angiosarcoma who underwent radical surgical resection after a prolonged response to weekly docetaxel and complementary radiotherapy.

**Conclusion:**

Combined treatment with weekly docetaxel and radiotherapy may be a valid alternative for the treatment of locally advanced cardiac angiosarcoma; the combination can lead to radical surgical resections, avoiding the cumulative cardiotoxicity of antracycline-based regimens.

## Background

Primary cardiac neoplasms are extremely rare, with an incidence of approximately 0.0001–0.030%. Although 75% of these tumors are benign, the remaining 25% are malignant, and angiosarcoma is the most frequent histotype.

Males are affected more often than females in a ratio of 2–3:1, and these neoplasms occur more commonly in individuals younger than 65 years. The mean survival time is 4 months, and most patients die within few months of diagnosis.

From a clinical point of view, primary cardiac neoplasms frequently occur with non-specific symptoms, such as dyspnea, weight loss, fatigue, anemia, and chest pain. In most cases, an early diagnosis is difficult. In addition to physical examination, electrocardiogram (ECG), cardiac catheterization and imaging studies such as computed tomography (CT), nuclear magnetic resonance (NMR) and echocardiography are the gold standard for diagnosis. Moreover, transthoracic echocardiography can evaluate the location, shape, size and mobility of the mass. Transesophageal echocardiography provides information about the insertion point of the tumor. Cardiac NMR is a more specific and sensitive tool than CT scan for diagnosis. NMR evaluation can determine whether the angiosarcoma is hemorrhagic or necrotic. Endomyocardial biopsies are usually negative, so a surgical exploration and intraoperative frozen sections are strongly suggested.

Current management of cardiac angiosarcoma, although still controversial, uses a multimodality approach, combining surgery, chemotherapy and radiotherapy. Radical resection followed by adjuvant radiotherapy represents the standard treatment in cases of limited disease, although the majority of patients present with locally advanced or metastatic disease [1, 2]. In case of locally advanced disease, integrated treatment with chemotherapy and radiotherapy may lead to shrinkage of the mass, allowing for radical surgery. Doxorubicin-based regimens are the gold standard treatment for locally advanced and metastatic angiosarcoma; however, the known cardiotoxicity of the anthracyclines can limit their use, especially in patients with intracardiac disease [3]. Taxanes alone or in combination with gemcitabine have shown high antitumor activity in angiosarcoma, although they generally have limited activity in soft tissue sarcomas. According to these data, taxanes are considered an alternative option for angiosarcoma. Until now, little evidence has been accumulated about the activity of taxanes in metastatic or locally advanced angiosarcoma [4–7]. We report the case of a Caucasian 23-year-old man with locally advanced cardiac angiosarcoma who underwent radical surgical resection after a prolonged response to weekly docetaxel and complementary radiotherapy.

## Case presentation

**Fig. 1:**
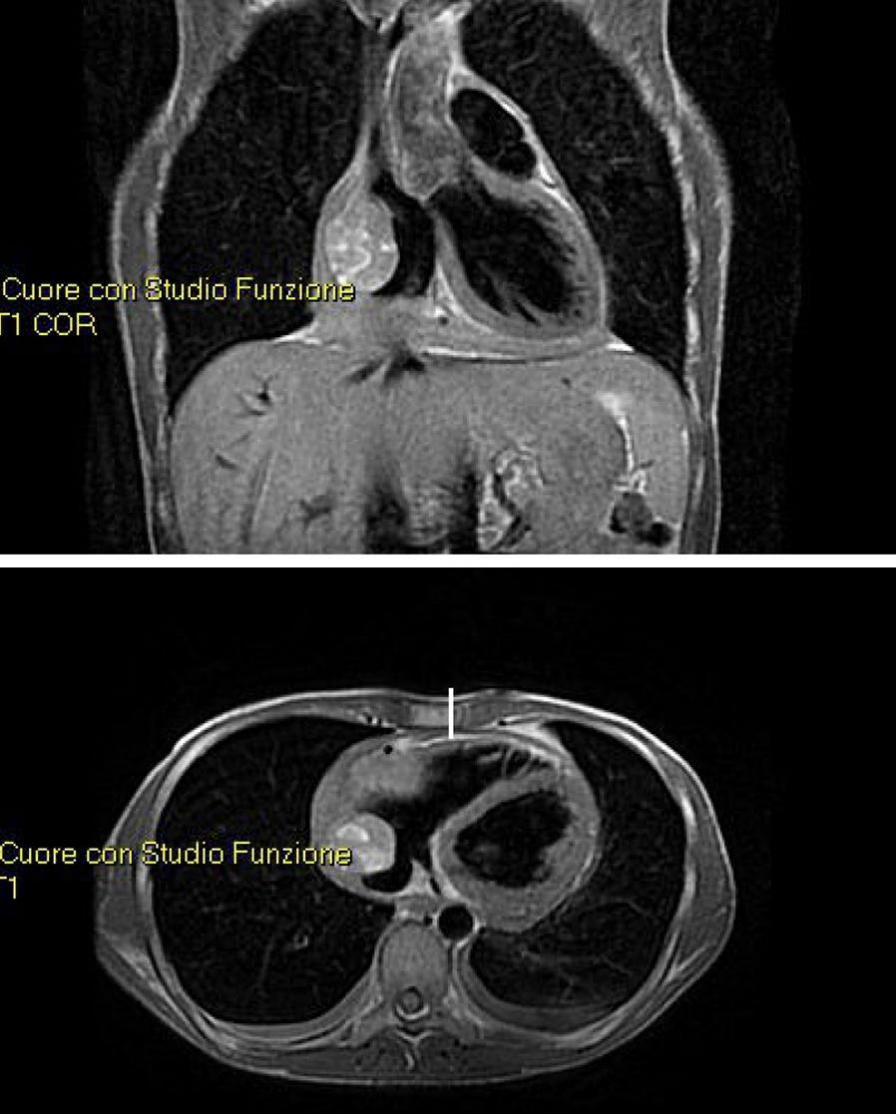
Nuclear magnetic resonance imaging before neoadjuvant therapy with docetaxel.

In April 2008, a healthy Caucasian 23-year-old man went to the emergency room for the sudden onset of dyspnea on exertion, fatigue, chest discomfort, fever, and night sweats. Upon physical examination, his vital signs were stable, with only mild tachycardia (heart rate 96/min). There were no palpable lymph nodes. Jugular venous pressure, carotid upstroke, and heart sounds were not clinically relevant. There were no appreciable murmurs. Respiratory and abdominal objective examinations were normal. A transthoracic echocardiogram revealed the presence of a profuse pericardial effusion; therefore an evacuative pericardiocentesis was performed, draining approximately 900 mL of serum blood material. A subsequent chest CT scan showed a solid and expansive mass, about 8.4 cm in diameter with a partial endocavitary development in the right atrium, associated with multiple mediastinal adenopathies and three noncalcified pulmonary micronodules. An NMR scan confirmed the previous CT findings (Fig. 1), and a subsequent CT- Positron Emission Tomography (PET) scan showed high glucose uptake in the mass (max standardized uptake value—SUV = 6.5). The histological examination performed by multiple biopsies of the lesion revealed a malignant mesenchymal neoplasm compatible with angiosarcoma.

**Fig. 2:**
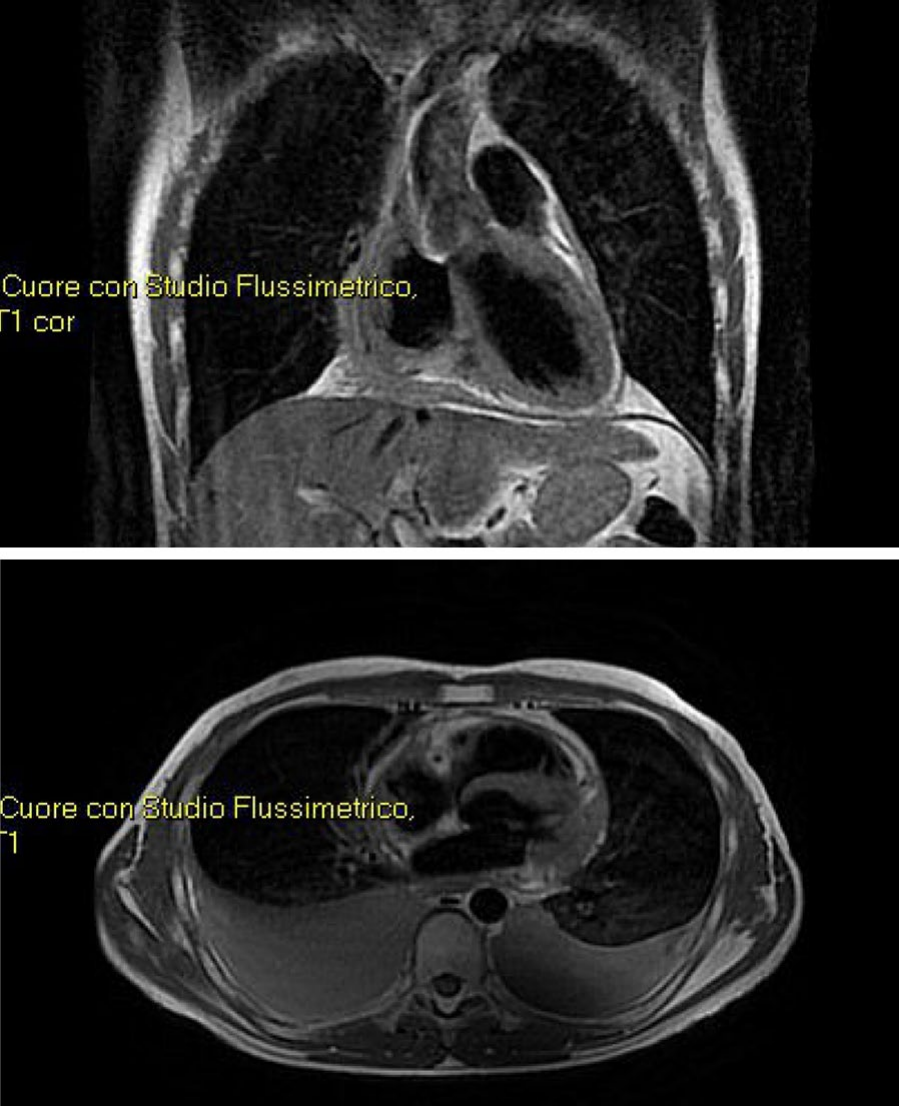
Nuclear magnetic resonance imaging after neoadjuvant therapy with docetaxel (24 administrations).

Surgical treatment with radical intent was initially excluded, given the size of the mass, its location close to the coronary ostium and the suspicion of extracardiac involvement. Therefore, a combined radiochemotherapeutic neoadjuvant treatment was planned. In order to preserve myocardial function as much as possible to allow for a subsequent surgical resection, doxorubicin was avoided, and first-line treatment with docetaxel was chosen. From June 2008 to September 2008, the patient was treated with four cycles of weekly docetaxel (35 mg/mq) with excellent tolerance. The subsequent NMR revaluation showed the disappearance of pulmonary nodules and a reduction of the atrial mass by about 1 cm. Subsequently, percutaneous conformal radiotherapy at a total dose of 5,400 centigray (cGy), with a daily fractionation of 180 cGy was performed, followed by 13 additional infusions of weekly docetaxel at the previous dose. Although the radiological revaluation confirmed a further reduction in size of the cardiac mass and a reduction in size of mediastinic adenopathies, the surgical resection was still excluded. Therefore the patient continued chemotherapy, receiving a total of 24 cycles of weekly docetaxel, until May 2009. Since a further reduction of 1.2 cm in size of the atrial mass was found by NMR (Fig. 2) and CT-PET scan, the surgical treatment was performed in July 2009. After surgery, the patient was treated again with weekly docetaxel for a total of 10 cycles in the adjuvant setting with overall good tolerance. A follow-up radiological revaluation (NMR, PET) did not show any signs of recurrence. A close follow-up was set during which several evacuative thoracenteses were performed because of a recurrent bilateral neoplastic pleural effusion until the patient died in April 2010 of respiratory failure about 2 years after diagnosis. The last radiological assessment showed progressive disease with pleural and liver metastasis.

## Discussion

Doxorubicin-based chemotherapy represents the standard medical treatment of cardiac angiosarcoma, even if the long-term benefit of chemotherapy and/or radiotherapy is still unsettled. However, given the high tumor aggressiveness, a multimodal approach seems to be favoured. Several studies reported survival times ranging from 12 to 30 months with the use of various combinations of surgery, chemotherapy, radiation and/or transplant in cases of locally advanced or metastatic disease [8]. In case of limited disease, after radical resection, a multidisciplinary approach with adjuvant chemotherapy and radiotherapy may improve overall survival [9, 10].

At present, chemotherapeutic regimens for cardiac angiosarcoma are the same as for all soft tissue sarcomas. Currently, the standard treatment for metastatic or locally advanced cardiac angiosarcoma is doxorubicin, even if the benefit in terms of survival is modest (progression-free survival of about 5 months).

The role of radiotherapy in combination with chemotherapy is still controversial in cases of locally advanced disease, given the high cumulative risk of cardiotoxicity due to the potential radiation-induced cardiotoxicity added to the known cardiotoxicity of anthracycline-based therapy. High doses of radiation to improve control of local tumor increase the risk of severe adverse events.

The most effective method to improve the outcome of cancer radiation therapy is to concentrate the dose of radiation only on the tumor. This improves local tumor control and also reduces adverse events [6].

Given the promising results showing high activity of taxanes in angiosarcoma, some evidence on the efficacy of taxanes in cardiac angiosarcoma has recently been accumulated. Taxanes can be used as radiosensitizer agents to decrease the adverse effects of high-dose standard fractionated radiotherapy [6]. In 2008, Nakamura et al. noted a durable response of 12 months using radiation with concomitant and then maintenance docetaxel [7]. In a case report in 2011, Suderman et al. observed a partial response with a progression-free survival of 16 months [6]. A good response has also been obtained using concomitant radiochemotherapy with carboplatin and paclitaxel [5].

Similar to the above data, our case report supports the use of weekly docetaxel as first-line treatment for locally advanced primary cardiac angiosarcoma, suggesting its efficacy especially when the goal is radical surgical resection, which represents the most important factor affecting the prognosis of cardiac angiosarcoma, which otherwise is inevitably unfavourable.

Despite these data, the underlying mechanism of action of taxanes in angiosarcoma remains unclear. It is already known that the taxanes are also effective in patients with Kaposi’s sarcoma, another vascular-derived tumor. In vitro, microtubule-targeted drugs exhibit potent antiangiogenic activity at a low concentration. Pasquier et al. characterized two distinct effects of paclitaxel on human endothelial cells: cytostatic effects at low concentrations and cytotoxic effects at high concentrations. The cytostatic effect is associated with increased mitochondrial membrane potential, p53 overexpression, and modification of the Bax/Bcl-2 ratio, whereas the cytotoxic effect involves inhibition of endothelial cell proliferation without inducing apoptosis and without any structural modification of the microtubule network [11]. Therefore, it can be assumed that taxanes may also act as antiangiogenic agents in cardiac angiosarcoma.

## Conclusion

The overall prognosis for patients with cardiac angiosarcoma is generally poor due to its local aggressiveness and its early metastatic potential. Until now, surgical resection seems to provide the best chance for prolonging survival. Even if doxorubicin-based chemotherapy remains the treatment of choice in unresectable and metastatic soft tissue sarcoma or locally advanced cardiac angiosarcoma, this regimen may be associated with a high risk of cumulative cardiotoxicity, resulting in less chance of radical surgery.

Our case report would suggest that combined treatment with weekly docetaxel and radiotherapy may be a valid alternative for the treatment of locally advanced cardiac angiosarcoma. This combination can lead to radical surgical resection, avoiding the cumulative cardiotoxicity of anthracycline-based regimens.

## Abbreviations

ECG: electrocardiogram
CT: computed tomography
NMR: nuclear magnetic resonance
PET: positron emission tomography
cGy: centigray
SUV: standardized uptake value
